# Walking improvement in chronic incomplete spinal cord injury with exoskeleton robotic training (WISE): a randomized controlled trial

**DOI:** 10.1038/s41393-022-00751-8

**Published:** 2022-01-29

**Authors:** Dylan J. Edwards, Gail Forrest, Mar Cortes, Margaret M. Weightman, Cristina Sadowsky, Shuo-Hsiu Chang, Kimberly Furman, Amy Bialek, Sara Prokup, John Carlow, Leslie VanHiel, Laura Kemp, Darrell Musick, Marc Campo, Arun Jayaraman

**Affiliations:** 1 Moss Rehabilitation Research Institute, Elkins Park, PA USA; 2 School of Medical and Health Sciences, and Exercise Medicine Research Institute, Edith Cowan University, Joondalup, WA Australia; 3 Kessler Foundation, West Orange, NJ USA; 4 Icahn School of Medicine at Mount Sinai, New York, NY USA; 5 Courage Kenny Research Center-Allina Health, Minneapolis, MN USA; 6 Kennedy Krieger Institute, Baltimore, MD USA; 7 John Hopkins School of Medicine, 733 N Broadway, Baltimore, MD 21205 USA; 8 Department of Physical Medicine and Rehabilitation, McGovern Medical School, The University of Texas Health Science Center at Houston, Houston, TX USA; 9 NeuroRecovery Research Center at TIRR Memorial Hermann, Houston, TX USA; 10 Marianjoy Rehabilitation Hospital, Wheaton, IL USA; 11 Burke Neurological Institute, White Plains, NY USA; 12 Shirley Ryan AbilityLab, Chicago, IL USA; 13 Discovery Statistics, Orange County, CA USA; 14 Ekso Bionics Ltd, Richmond, CA USA; 15 Kemp Clinical Consulting Co. LLC, Carlsbad, CA USA; 16 Mercy College, Dobbs Ferry, NY USA

**Keywords:** Rehabilitation, Randomized controlled trials

## Abstract

**Study design:**

Clinical trial.

**Objective:**

To demonstrate that a 12-week exoskeleton-based robotic gait training regimen can lead to a clinically meaningful improvement in independent gait speed, in community-dwelling participants with chronic incomplete spinal cord injury (iSCI).

**Setting:**

Outpatient rehabilitation or research institute.

**Methods:**

Multi-site (United States), randomized, controlled trial, comparing exoskeleton gait training (12 weeks, 36 sessions) with standard gait training or no gait training (2:2:1 randomization) in chronic iSCI (>1 year post injury, AIS-C, and D), with residual stepping ability. The primary outcome measure was change in robot-independent gait speed (10-meter walk test, 10MWT) post 12-week intervention. Secondary outcomes included: Timed-Up-and-Go (TUG), 6-min walk test (6MWT), Walking Index for Spinal Cord Injury (WISCI-II) (assistance and devices), and treating therapist NASA-Task Load Index.

**Results:**

Twenty-five participants completed the assessments and training as assigned (9 Ekso, 10 Active Control, 6 Passive Control). Mean change in gait speed at the primary endpoint was not statistically significant. The proportion of participants with improvement in clinical ambulation category from home to community speed post-intervention was greatest in the Ekso group (>1/2 Ekso, 1/3 Active Control, 0 Passive Control, *p* < 0.05). Improvements in secondary outcome measures were not significant.

**Conclusions:**

Twelve weeks of exoskeleton robotic training in chronic SCI participants with independent stepping ability at baseline can improve clinical ambulatory status. Improvements in raw gait speed were not statistically significant at the group level, which may guide future trials for participant inclusion criteria. While generally safe and tolerable, larger gains in ambulation might be associated with higher risk for non-serious adverse events.

## Introduction

In the chronic phase after incomplete spinal cord injury (iSCI), those individuals with residual gait function, may improve walking function by engaging in an intensive gait training regimen [1–3]. Rehabilitation robotic exoskeletons can readily deliver a participant-specific and precise high-dose training regimen, and may simultaneously reduce the physical stress imposed on therapists, relative to conventional gait training strategies, such as manually assisted stepping practice via harness and treadmill. Exoskeleton training is predicted to improve walking function in participants receiving usual care, but not expected to be superior to intensity-matched manual training, or other labor-intensive gait training strategies. The rationale to implement exoskeleton robotics as a preference in gait training is based on precision dosing, overground (OG) training, and reduced therapist burden for high-repetition training. We consider that robotic exoskeleton gait training in SCI is cost effective [4], not to replace the skilled human operator and clinical decision-maker, but rather to offset the heavy manual labor requirement that continues to be a substantial and under-reported occupational risk for therapists [5–7].

Furthermore, clinical research related to OG robotic exoskeletons has been mostly limited to safety and tolerability trials or single-arm clinical studies [8–11], or randomized trials assessing device-dependent gait function [12]. There are currently no randomized controlled clinical trials comparing the impact of robotic exoskeleton OG training vs. conventional gait training strategies on independent gait function in individuals with chronic iSCI.

The primary objective of the present study was to demonstrate that an OG robotic exoskeleton-based 12-week gait training regimen, can lead to a clinically meaningful improvement in robot-independent walking speed.

## Method

### Study design

This was a randomized, prospective, multi-center, assessor-blind, longitudinal, comparative study to evaluate the efficacy of robotic exoskeleton gait training versus standard gait training or usual care. The practice schedule followed a prior robotic rehabilitation clinical trial [13], while the treatment session structure and progression were developed by the investigators.

Prior to the randomization phase, each site was required to enroll one to three participants in a run-in phase. The run-in phase was designed to (1) train and carefully supervise the sites prior to starting the randomization (2) test the recruiting ability of the selected sites (3) test the assessment time-points for primary and secondary endpoints, and (4) ensure that the inclusion/exclusion criteria were adequate. As the run-in phase was intended for study-specific training and participants were not randomized, these data were not included for analysis.

Participants could volunteer for the study if they had motor incomplete upper motor neuron paraplegia or tetraplegia, from traumatic or non-traumatic injury at least one year prior, and self-selected gait speed of <0.44 meters/second (m/s) with the ability to take at least one step (see Table 1 for full list of inclusion and exclusion criteria). Study participants were recruited from outpatient clinics and advertisement to local spinal cord injury organizations, following Institutional Review Board (IRB) approval at each site. Informed consent was obtained for all participants.

**Table 1:** WISE trial inclusion and exclusion criteria.

Inclusion criteria	Exclusion criteria
1. Motor incomplete paraplegia or tetraplegia, chronic (> 1 year after the injury). Non-traumatic SCI injuries can be included, given they are neurologically stable conditions for 12 months (e.g. tumor, transverse myelitis, but NOT Guilliane-Barré) 2. NLI C1- approximately T10 (inclusive, for upper motor neuron injuries only), as determined by the International Standards for Neurological Classification of SCI (ISNCSCI) 3. Sufficient diaphragmatic strength such that respiration is not compromised with exercise. 4. Sufficient upper extremity strength to use a front wheeled walker by manual muscle testing (minimum triceps strength bilaterally of 3/5, shoulder abduction and flexion/extension 4/5) 5. AIS-C SCI & AIS-D SCI, as determined by the International Standards for Neurological Classification of SCI (ISNCSCI) 6. Ambulates at a self-selected speed of <0.44 m/s with or without physical assistance and assistance device 7. Able to advance at least one leg forward (volitionally with lower extremity movement (not as a result of trunk movement or spasticity) while using parallel bars, walker or crutches, with or without braces, and up to 2 people to assist with safety and balance only. Stepping is to be performed by the patient (without PT assistance at the lower extremities and no BWS). 8. 18–75 yrs, inclusive 9. No current or history of other neurological conditions 10. Screened and cleared by a physician 11. Involved in standing program or must be able to tolerate at least 15 min upright without signs or symptoms of orthostatic hypotension 12. Weigh 220 pounds (100 kg) or less 13. Be able to fit into the Ekso device 14. Between approximately 5′0″ and 6′4″ (1.5 m and 1.9 m) tall 15. Standing hip width of approximately 18″ (45 cm) or less 16. Have near-normal range of motion in hips, knees, and ankles	1. AIS-A SCI or AIS-B SCI 2. Lower motor neuron injuries, as shown by absent reflexes during bilateral quadriceps and Achilles tendon taps 3. < 3 months since previous intensive gait training regimen, FES cycling program, and/or lower extremity Botox injections. The gait training regimen was meant to be formal gait training with feedback for progression of walking (i.e. PT sessions). Participant could have a regular home exercise program and/or a walking exercise program with a companion/ trainer for safety, but not for verbal or tactile cues or feedback regarding gait in the 3 months before initiating the protocol. If participant had a home exercise program and/or a walking exercise program, these programs (except FES cycling) could be continued without changes throughout the protocol. Electrical stimulation devices used regularly for foot drop during ambulation could be considered a brace and could continue to be used as usual throughout the protocol. Upper extremity Botox injections were permissible before and during the protocol. One or two PT sessions were allowed to obtain a new brace or progress bracing and check for fit and safety, but no sustained gait training could occur. 4. Already walking at self-selected ambulation speeds of at least 0.44 m/s with or without assistance 5. Currently involved in another intervention study 6. Concurrent neurological disease 7. Hip flexion contracture greater than ~17° 8. Knee flexion contracture greater than 12° 9. Unable to achieve neutral ankle dorsiflexion with passive stretch (neutral with max 12° knee flexion) 10. Leg length discrepancy a. Greater than 0.5″ (1.27 cm) for upper leg b. Greater than 0.75″ (1.91 cm) for lower leg 11. Spinal instability 12. Unresolved deep vein thrombosis 13. Uncontrolled autonomic dysreflexia 14. Severe muscular or skeletal pain 15. Spasticity that prevents joint motion (severe stiffness or rigidity,) where both legs have a MAS score of 3 or higher for half or more of their proximal lower extremity muscles; proximal muscles include hip flexors/extensors/adductors and knee flexors/extensors. 16. Open skin ulcerations on buttocks or other body surfaces in contact with exoskeleton or harness 17. Pregnancy 18. Cognitive impairments – unable to follow 2 steps commands and communicate for pain or to stop session 19. Shoulder extension ROM < 50° excludes crutches during sit to stand or vice versa. (Walking with crutches permitted.) 20. Participant requires the assistance of more than one therapist to transfer safely. 21. Uncontrolled or severe orthostatic hypotension that limits standing tolerance; defined as sustained, symptomatic drops in systolic and diastolic blood pressure when moving from sitting to standing 22. Active heterotrophic ossification (HO), hip dysplasia, or hip/knee axis abnormalities 23. Colostomy 24. History of long bone fractures since the SCI, secondary to osteoporosis 25. Unable to sustain current medication regimen 26. Any reason the physician may deem as harmful to the participant to enroll or continue in the study

### Intervention

#### Run-in participants

One to three participants per site were required to complete the Ekso intervention protocol as run-in participants, to ensure that assessment and training procedures were practiced and followed as required in the clinical trial protocol.

Following the satisfactory completion of at least one set of midpoint assessments during the run-in phase, subsequent *main study* participants (a separate cohort) were enrolled and randomly assigned to one of three study arms via a computer-generated allocation table. These study arms included Ekso Robotic Intervention, Active Control or Passive Control (2:2:1 randomization ratio, respectively).

#### Ekso intervention

**Fig. 1:**
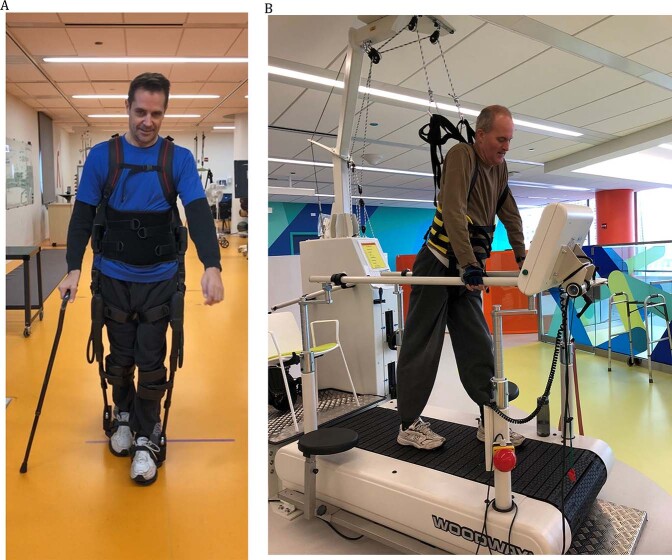
Electromechanical devices used for the active intervention groups. **A** Photograph with permission, showing an SCI participant training in the robotic exoskeleton suit. **B** Photograph with permission, showing an SCI participant from the Active Control group training using the body weight supported treadmill prior to overground stepping practice.

Ekso GT robotic gait training (3x/wk, 12 weeks, 36 sessions) sessions comprised 45 min of gait training in the Ekso device (minimum 300 steps per session, Fig. 1), and if possible, OG training without body weight support (BWS). The 45 min excluded set-up/donning/doffing time and included standing/up time, walking time, and seated rest breaks. Standard OG gait training was introduced when the participant required only minimal assistance of one therapist and one aide to help control the assistive device. This was assessed weekly during the 10-meter walk test (10MWT) performed every 3rd session. At that point, sessions consisted of 30 min of session time gait training in the Ekso, followed by 15 min of session time performing standard OG gait training outside the Ekso, for a total of 45 min. See Table 2A for the Ekso training timing, settings and progression strategy. In brief, the 15-min OG gait training could take place at the first session if the criterion for assistance was met. During the 15-min OG gait training, any intervention, device, or bracing could be used (except Ekso, Body Weight Supported Treadmill Training, BWSTT, or any BWS). Stairs could be included during this OG gait training for a maximum of 5 min per session when the participant was able to perform them with only minimal assist or less of one physical therapist (PT).

**Table 2:** (A) WISE trial Ekso training progression strategy. (B) WISE trial Active Control training progression strategy.

**(A)**				
**General rules for Ekso Active Group:**				
(1) Sessions are divided into three 15-min segments. This includes any rest breaks required, as well as a 5-min warm-up and 2-min cool down per session required by week 2.				
(2) Sessions begin with a goal of **a minimum of 300 steps per session in Ekso within trajectory**. Then can do outside of trajectory.				
(3) All sessions after session #3 must include a 5-min warm-up and end with a 2-min cool down in Bilateral/Adaptive.				
(4) All exoskeleton and assistive device progressions should be done in Bilateral/Adaptive. (Examples: FRW to Crutches; step length increase; step height decrease; target adjustment; turning mode or technique).				
(5) Each leg may be considered individually when considering reducing Fixed assist level, or choosing high/low assistance/resistance in 2Free. No unilateral trajectory-free stepping is allowed to avoid promoting gait asymmetries.				
(6) **If the participant is exceeding 750 steps per session, the therapist should increase the challenge to the participant**.				
(7) Excluding Ekso donning and doffing time, each session will last a total of 45 min, which will include standing/up time, walking time, and seated rest breaks.				
(8) Overground walking will be included when the participant requires only minimal assistance of one therapist and the assistance of one aide to control the assistive device for at least 10 m.				
(9) If participant is not yet performing overground gait training, all 45 min of session will be done in Ekso.				
(10) If participant is performing overground gait training, session will consist of 30 min of gait training in the Ekso, followed by 15 min performing overground gait training for a total of 45 min.				
(11) Step monitors are to be used during any OG gait training.				
**Timeline and settings**	**Assist**	**Swing assist**	**Progression and adjustments**	**Considerations**
Sessions 1–3	Bilateral	Trajectory controlled: adaptive	Pre-gait weight shifting Stance support remains at “Full” Minimize upper extremity loading. Optimize step height; swing time; step length; targets; etc.	Balance and gait progression Consistently hitting >300 steps per session Adjust targets/swing time/step length as appropriate
Anytime session 3+	Bilateral	Trajectory controlled: adaptive	Pre-gait weight shifting when needed. Once participant has consistently managed >300 steps/session, progress to crutches if appropriate and encourage minimal UE loading	Adjust targets/swing time/step length as appropriate Step count with crutches should be at least 80% of step count with walker.
Training guidelines	Bilateral	Trajectory controlled: adaptive to fixed Trajectory free: 2Free	PT may progress the participant by lowering the fixed assist for each leg, as tolerated and clinically appropriate. Stance support may be changed from “Full” to “Flex”. Once initial 300 steps in trajectory are completed, PT may progress the participant via trajectory-free stepping using “2Free”. Stance support should begin at appropriate level. As participant improves stance control, support may be reduced as tolerated and clinically appropriate. Swing support should be assessed at “neutral”. If a leg is not able to complete a step, “high”/”low assistance” may be provided for more normalized stepping. If a leg is stepping far outside of the general trajectory, “high”/”low resistance” may be provided for more normalized stepping. Progress to more symmetrical gait.	Step count must be at least 300 within trajectory. Must include 5 min warm-up at beginning and 2 min cool down at end in bilateral adaptive. Borg range 12-17 to prevent fatigue early in the session No more than 3 swing completes per minute in Fixed assist. (If so, then increase Fixed swing assist by 10 or reduce swing complete time) No unilateral trajectory-free stepping to avoid promoting gait asymmetries.
Progression guidelines	Bilateral	Trajectory controlled: adaptive to fixed Trajectory free: 2Free	If initial 300 steps within trajectory is achieved/projected, the therapist may challenge the participant by the following, as tolerated and clinically appropriate: Lower the Fixed assist bilaterally as appropriate Stance support may be changed from “Full” to “Flex”. Once initial 300 steps in trajectory are completed, set swing support at appropriate assistance/resistance for an appropriate clinical challenge	Step count must be at least 300 within trajectory. Must include 5 min warm-up at beginning and 2 min cool down at end in bilateral adaptive. Borg range 12-17 to prevent fatigue early in the session No more than 3 swing completes per minute in Fixed assist (If so, then increase Fixed swing assist by 10 or reduce swing complete time) No unilateral trajectory-free stepping to avoid promoting gait asymmetries.

**(B)**			
**General rules for Active Controls:**			
(1) Sessions are divided into three 15-min segments. This includes any rest breaks required, as well as a 5-min warm-up and 2-min cool down per session required by week 2.			
(2) Participants will perform gait training with BWSTT for the full session (all 3 segments) until the OG criterion is met. This gait training must continue for a **minimum of 300 steps** at the beginning of each session.			
(3) Manual assistance from the physical therapy team to facilitate normal stepping kinematics is permissible.			
(4) BWS and speed are to be determined by the physical therapist based on appropriate stepping kinematics, level of challenge to the participant, and safety of the participant and trainer(s).			
(5) Participants will perform gait training with BWSTT for the full session (all 3 segments) until the OG criterion is met. Participants may proceed to overground gait training **without BWS** only when they require minimal physical assistance of the physical therapist, plus assistive device control or supervision of another team member for at least 10 m.			
(6) Once the OG criterion is achieved:			
a. If the initial 300 steps **are not completed** by the end of the first segment, the middle 15-min segment must be gait training in BWSTT, and the final 15-min segment must be OG gait training.			
b. If the initial 300 steps **are completed** by the end of the first segment, the middle 15-min segment can be continued gait training in BWSTT or OG gait training, per PT choice. The final 15-min segment must be OG gait training.			
(7) Step monitors are to be used during the full 45 min= of gait training.			
	**Focus**	**Progression and adjustments as tolerated**^**a**^	**Considerations**
Sessions 1–3	• Determine parameters for best kinematics • Participant familiarization • Posture	Determine comfortable BWS, stepping range of speeds, amount of physical assistance at each location, and bout length	Educate and engage posture and basic stepping Educate and ensure joint protection
Sessions 4–6	• Posture • Good stepping kinematics • Increase load as tolerated^b^ • Increase range of speeds as tolerated All sessions should have 5-min warm-up and 2-min cool down.	• Decrease BWS if tolerated • Increase/decrease speed • Increase bout lengths	Engage posture and both swing/stance phases of stepping Ensure good posture, stepping kinematics, and joint protection. Borg range 12–17 to prevent fatigue early in the session
Sessions 7–18	• Increase load weekly if tolerated^b^ • Increase range of speeds as tolerated • Increase independence • Increase endurance All sessions should have 5-min warm-up and 2-min cool down.	• Decrease BWS if tolerated • Increase/decrease speed • Decrease physical assistance • Increase bout lengths, decrease rest breaks • Introduce walking sideways, backwards, stepping over obstacles, quick speed changes, quick start/stops if tolerated	Engage hip control and motor control (concentric/eccentric) of stepping, arm-swing Ensure good posture, stepping kinematics, and joint protection. Borg range 12–17 to prevent fatigue early in the session
Sessions 19–36	• Increase load weekly if tolerated^b^ • Increase range of speeds by as tolerated • Increase independence • Increase endurance • Increase adaptability All sessions should have ~5-min warm-up and ~2-min cool down.	• Decrease BWS if tolerated • Increase/decrease speed • Decrease physical assistance • Increase bout lengths, decrease rest breaks • Continue or introduce walking sideways, backwards, stepping over obstacles, quick speed changes, quick start/stops	Engage motor control of posture, hips, symmetric stepping, arm swing (when evaluable) Ensure good posture, stepping kinematics, and joint protection. Borg range 12–17 to prevent fatigue early in the session

*BWS* body weight support, *BWSTT* Body Weight Supported Treadmill Training, *OG* overground.

^a^PT can adjust one or multiple parameters at a time. PT can adjust parameters for interval training, e.g. lower BWS for 5 min.

^b^Training intensity should be increased first by increasing loading. If amount of loading puts participant or trainers at risk for injury, then increasing range of speeds or independence can be the focus of increasing intensity.

#### Active Control

The practice schedule was matched for the Active Control group, with each session comprising 45 min of BWSTT, and if possible, OG training without BWS. The 45 min excluded set-up/harnessing time and included standing time, walking time, and seated rest breaks. Sessions began with a minimum of 300 steps during BWSTT. Commencement criteria of OG training matched the Ekso group, and once achieved: (1) If the initial 300 steps were not completed by the end of the first segment, the middle 15-min segment was required to be gait training in BWSTT, and the final 15-min segment was required to be OG gait training, (2) If the initial 300 steps were completed by the end of the first segment, the middle 15-min segment could be continued gait training in BWSTT or OG gait training, per PT choice. The final 15-min segment was required to be OG gait training. See Table 2B for the BWSTT training progression strategy. The OG gait training took place at the first session if the criterion for assistance was met. During OG gait training, the same rules applied in this group as for the Ekso group. The Active Control training protocol was based on standard clinical practice guidelines at participating sites, with emphasis on task-specific training focusing on intensity and dose.

#### Passive Control

Participants in this group continued with daily activities as normal over 12 weeks. No new gait training, mobility therapy, nor new medications related to the condition under study, were to be commenced during the study period. Participants in this group came to the study sites for evaluations at baseline, 6 and 12 weeks. Participants randomized to this Passive Control group were offered Ekso Active or Active BWSTT sessions at the conclusion of their 12-week participation.

### Outcome measures

Demographic and other outcome measures were evaluated by blinded ASIA Impairment Scale (AIS) trained physical therapists. The primary endpoint of this study was change in gait speed (m/s) demonstrated during the 10MWT after the 12-week intervention (36 sessions), relative to baseline. Additional assessments were performed at the intervention period midpoint (6 weeks), and 12 weeks post-intervention for the active intervention groups. Both self-selected and fast speeds were performed. For each, the average of two trials was used for analysis. The number of participants who achieved the Minimal Clinically Important Difference (MCID) of 0.15 m/s [14] and the number of participants who transitioned from exercise or household ambulation (defined as self-selected walking speed of ≤0.44 m/s) to limited community or full community ambulation (>0.44 m/s) [15] during the self-selected speed gait assessment were also reported.

Secondary outcome measures included the Timed Up and Go (TUG) for functional mobility and balance (seconds), the 6-min walking test for endurance (meters), and the WISCI-II score (0–20 scale) for need of assistance and devices.

A dedicated Adverse Event (AE) and Serious Adverse Event (SAE) set of forms was used to track safety outcomes. Throughout the training period, an independent Data and Safety Monitoring Board (DSMB) assessed and adjudicated all SAEs and protocol violations. An independent study monitor reviewed trial and data quality at each site, during the run-in and final trial phase.

For comparison of therapist manual burden between active interventions, we employed the NASA-Task Load Index (TLX). The TLX is a widely used, validated measure of self-reported workload [16]. The instrument assesses perceived mental, physical, and temporal demands as well as effort, performance and frustration associated with a job task. Each dimension is rated on a visual analog scale (VAS) ranging from 0 to 20. The VAS is anchored using the terms ‘low’ and ‘high’.

### Sample size determination

The present study terminated early for reasons independent of trial findings as there was no interim analysis before stopping, rather the financial capability of the sponsor led to cessation of trial funding during the third year, and thus our results are unlikely to introduce bias by stopping [17]. Our analyses took place with the available data.

### Statistical analyses

This study design involved outcome variables measured on binomial and polynomial as well as continuous scales. For the continuous outcome variables, the general situation applied analysis of variance and covariance when there were *g* distinct groups with a sample of observations for each group. The general null hypothesis was that the outcome variable distribution was the same for the Ekso Group and the Active Control Group. The assumptions associated with the analysis of variance were: (1) the *g* samples were independent random samples, (2) the observations in group *i* (for each *i* = 1,2,…,g) were a random sample from a normal probability distribution with mean μ_*i*_ and variance σ_*i*_^2^, and (3) the *g* population variances, σ_1_^2^,….,σ_g_^2^, were equal to a common variance σ^2^. Inherent to these assumptions was the assumption of a linear additive model with equal sample sizes. However, since the sample size was below 30, this procedure could not be expected to give reliable probabilities and a normal distribution could not be assumed. Therefore, a *t* distribution was assumed [18].

A Student’s *t* test (paired sample) was used to compare the single group pre- and post-test means (i.e., 10MWT, Berg Balance Scale, 6MWT, and TUG). One-way analysis of variance techniques were used for multiple comparisons (i.e., Treatment Groups). In addition, when it was found that the variances were not homogeneous and the sampling distributions were not normal, non-parametric alternatives were employed. Those included the Wilcoxon–Mann–Whitney Rank Sum Test for two independent samples [19] and the Wilcoxon test for analysis of variance, which is appropriate for small sample sizes. Mean comparisons of repeated measures over time were performed using Tukey–Kramer HSD with alpha = 0.05. Within-group comparison refers to pre to post change for a single group, between-group comparison refers to comparison of change between groups.

Binomial and polynomial outcomes (i.e., Velocity Cutoff and MCID) were assessed using chi-square statistical tests of the hypothesis that the response rates were the same in each sample category. Correction for continuity, exact probabilities, and 95% confidence intervals were computed where appropriate. Univariate analysis with Fisher’s Exact Test was employed to analyze dichotomous outcomes such as safety endpoints.

All analyses were performed using JMP Statistical Software, Version 15.0 (SAS Institute Inc., Cary, NC).

## Results

**Fig. 2:**
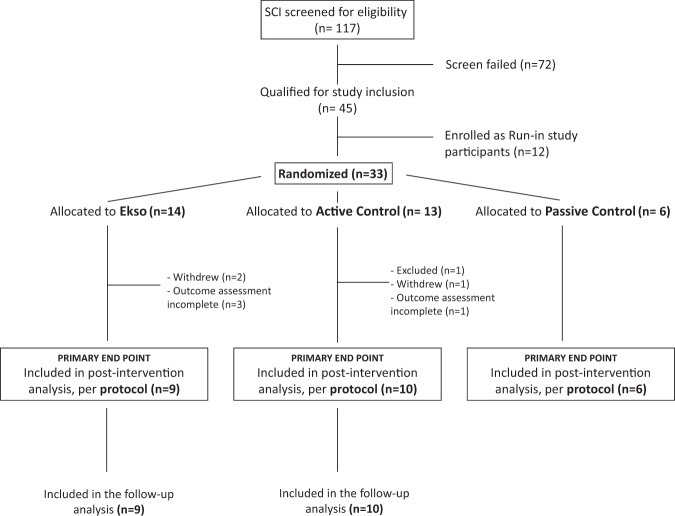
Study consort diagram.

Between September 26, 2016 and September 3, 2019, across seven US sites (6 main study, 1 run-in), 45 participants were enrolled, of which 33 were randomized to the main study and 12 enrolled as run-in participants (Fig. 2). Of the 33 randomized participants, 25 completed the assessments and training related to the primary endpoint analysis (per protocol); 9 Ekso, 10 Active Control, 6 Passive Control. Withdrawals were not intervention-related, and there were three SAEs described below. Baseline clinical characteristics for the sample population in each group are provided in Table 3. Clinical characteristics were statistically comparable for all treatment groups at baseline.

**Table 3:** Baseline clinical and demographic data for all enrolled participants: (A) Ekso group baseline clinical characteristics, (B) Active Control group baseline clinical characteristics, and (C) Passive Control group baseline clinical characteristics.

Subject ID	Gender	Age (yrs)	Time since injury (yrs)	AIS	ISNCSCI (motor) (L-R)	ISNCSCI (sensory) (L-R)	WISCI-II	SCI-FAI	SCIM-III	LEMS	UEMS	SCATS (clonus) (L-R)	SCI-SET Total Score	Gait speed (SS, m/s)
**(A)**														
01-123^a^	F	41	22.3	C	T1-T2	T2-C8	12	2	13	14	50	2-2	0.00	0.18
02-108^a,b^	M	26	7.8	C	L1	T10-L4	12	3	17	19	50	0-0	−0.11	0.34
02-115^a,b,c^	F	38	2.1	C	T11	T11	19	5	33	23	50	0-2	−1.00	0.41
02-127^1^	M	48	8.0	D	C3-C6	C3-C4	16	4	21	37	37	2-2	−1.20	0.22
02-130^a,b^	M	48	28.5	D	C6-T1	C7-C4	19	5	33	40	47	1-1	−0.63	0.43
02-132	M	37	2.3	D	C6-C7	C6-T8	6	1	7	28	38	1-1	−0.29	0.38
04-106^1^	F	35	9.3	C	T1	T1	9	3	17	20	50	1-1	−0.50	0.16
07-108^1^	M	58	7.2	D	C5-C7	C5-T10	13	3	16	22	28	1-1	0.00	0.34
07-110^a,b,c^	F	48	1.2	D	T1-C7	C4	12	4	30	45	48	1-0	−1.42	0.26
08-105^a,b,d^	F	46	8.1	C	C7	T2	13	3	20	26	32	1-1	−0.53	0.42
09-106	M	34	1.6	C	T7-T6	T7-T6	6	1	17	17	50	1-1	−0.35	0.09
09-108	M	41	2.4	D	C5-C3	C5-C3	8	2	6	36	32	2-2	−1.60	–

**(B)**														
01-115^a,d^	F	61	–	C	L1–T10	L1-T10	16	4	19	7	50	0-0	−0.49	0.24
02-109^a,d^	F	28	2.8	D	T5-T7	T5-T7	19	4	31	42	50	0-0	−0.29	0.30
02-114^a^	F	34	10.4	C	L1	C6-L1	6	2	17	11	50	0-0	−2.63	0.09
02-118^a^	M	56	10.5	D	T1	C7-C6	19	4	25	24	50	1-1	−0.31	0.35
02-125^a^	M	56	8.8	C	C7–C6	C7-C6	13	2	5	23	28	0-0	−0.26	0.16
04-103^a^	M	65	2.3	D	T1	C8-T1	13	4	21	38	50	1-1	−0.12	0.30
04-104^a^	M	52	12.3	D	C3	C3	18	4	23	33	36	1-1	−0.41	0.26
07-107^a^	F	62	4.2	D	C1	C1	6	2	12	50	48	1-1	−2.47	0.16
08-103^a^	M	31	6.2	D	C1	C4-C8	13	4	19	39	50	0-0	−0.09	0.17
08-106	F	64	14.2	D	T12-L2	T12-L1	16	5	29	44	50	0-0	−0.22	0.48
09-104	F	43	3.6	D	C5	C4	13	1	4	36	14	2–2	−1.12	0.03
09-105^a,c^	M	71	2.1	D	C5	T7-T4	13	5	20	47	40	0-0	−0.44	0.35

**(C)**														
01-116^a^	M	74	1.7	D	C7	L4-L3	9	3	22	45	46	1-0	−0.14	0.17
02-112^a^	M	59	10.0	D	C7	C5	16	5	30	41	36	0-1	−0.94	0.34
02-129^a^	M	45	11.0	D	C3-C2	C3-C2	6	3	19	18	50	0-0	−0.56	013
07-105^a^	F	59	1.3	D	C3-C7	C3-T1	13	3	16	43	44	0-0	−0.09	0.33
08-104^a^	M	26	1.2	D	T4	T5-T4	13	2	19	35	50	1-1	−0.12	0.12
09-107^a^	M	31	1.0	D	C6–C7	T9	13	3	17	33	34	1-1	−0.23	0.24

Baseline clinical and demographic data for all enrolled participants in (A) Ekso, (B) Active Control and (C) Passive Control groups. Baseline characteristics were comparable across all treatment groups.

*AIS* ASIA Impairment Scale, *ASIA* (American Spinal Injury Association), *ISNCSCI* International Standard for Neurological Classification of Spinal Cord Injury, *WISCI-II* Walking Index for Spinal Cord Injury, *SCI-FAI* Spinal Cord Injury Functional Ambulation Index, *SCIM-III* Spinal Cord Independence Measure, *LEMS* Lower Extremity Motor Score, *UEMS* Upper Extremity Motor Score, *SCATS* Spinal Cord Assessment Tool for Spastic Reflexes, *SCI-SET* Spinal Cord Injury Spasticity Evaluation Tool, *SS* self-selected.

^a^Per Protocol Group.

^b^Responder to > 0.44  m/s threshold.

^c^Responder to MCID threshold.

^d^Responder to both thresholds.

### Gait speed (primary)

**Fig. 3:**
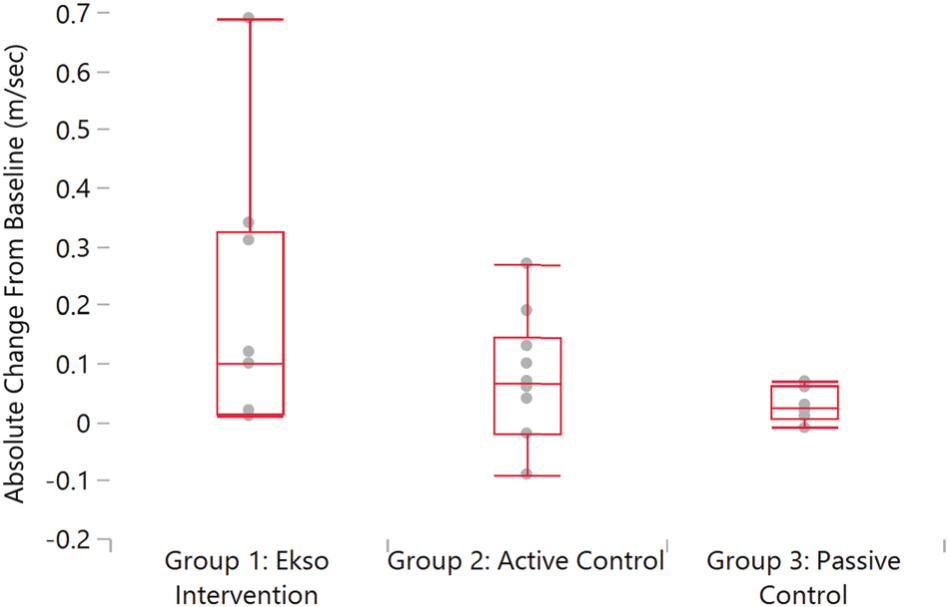
Change in gait speed post intervention relative to pre-intervention: Graphical representation of absolute change in gait speed (m/sec; meters/second).

*Self-selected* gait speed following the 12-week intervention increased in the Ekso group by 51% (mean, SD; 0.18 ± 0.23 m/s) Active Control by 32% (0.07 ± 0.11 m/s) and Passive Control 14% (0.03 ± 0.03 m/s), within group and between group comparisons *p* > 0.05 (Fig. 3, Table 4).

**Table 4:** Change in gait speed post intervention relative to pre-intervention: Statistics for absolute change in gait speed in m/s.

Quantiles							
Level	Minimum	10%	25%	Median	75%	90%	Maximum
Group 1: Ekso Intervention	0.01	0.01	0.02	0.10	0.33	0.69	0.69
Group 2: Active Control	−0.09	−0.08	−0.02	0.07	0.15	0.26	0.27
Group 3: Passive Control	−0.01	−0.01	0.01	0.03	0.06	0.07	0.07

Means and Std Deviations						
Level	Number	Mean	Std Dev	Std Err Mean	Lower 95%	Upper 95%
Group 1: Ekso Intervention	9	0.18	0.23	0.08	0.00	0.36
Group 2: Active Control	10	0.07	0.11	0.03	−0.00	0.15
Group 3: Passive Control	6	0.03	0.03	0.01	−0.00	0.06

Means Comparisons for all pairs (Tukey-Kramer HSD)						
Level	- Level	Difference	Std Err Dif	Lower CL	Upper CL	p-Value
Group 1: Ekso Intervention	Group 3: Passive Control	0.15	0.08	−0.06	0.36	0.181
Group 1: Ekso Intervention	Group 2: Active Control	0.11	0.07	−0.07	0.29	0.309
Group 2: Active Control	Group 3: Passive Control	0.04	0.08	−0.16	0.24	0.854

*Maximal* gait speed following the 12-week intervention increased in the Ekso group by 44% (0.20 ± 0.24 m/s) Active Control 50% (0.14 ± 0.18 m/s) and Passive Control 14% (0.03 ± 0.13 m/s) within group and between group comparisons *p* > 0.05.

The highest individual absolute speed improvement at both self-selected and fast speeds was seen in the Ekso group. There was a marginal effect of improving by repeated testing as seen in the Passive Control group.

Mean improvement in walking speed for both intervention groups at the follow-up visit were not statistically significant (*p* > 0.05).

Improvement above the MCID (0.15 m/s) [14] during the self-selected speed test was examined between groups, with the highest *responder* proportion in the Ekso group at 3 of 9 or 1/3 of participants, 2 of 10 or 1/5 in the Active Control, and 0 in the Passive Control group (between-group difference in proportions *p* > 0.05).

For proportion of change in clinical ambulation category, the highest proportion of *responders* was in the Ekso group at 5 of 9 participants, 3 of 10 of the Active Control improved ambulation category, while 0 in the Passive Control group changed (between-group difference in proportions *p* < 0.05, Table 5).

**Table 5:** Change in clinical gait speed category.

Self-selected speed averaged trials 1 and 2 (Per Protocol Population)						
		Velocity for home v community			MCID (∆ from baseline)	
Evaluation/Group	*n*	Median (IQR)	*P* < 0.44 m/s	*P* > 0.44 m/s	*P* < 0.15 m/s	*P* > 0.15 m/s
Baseline						
EKSO Group	10	0.30 (0.16–0.39)	100%	0%		
Active Control	11	0.24 (0.14–0.30)	100%	0%		
Passive Control	6	0.22 (0.13–0.33)	100%	0%		
Endpoint						
EKSO Group	9	0.46 (0.22–0.65)	44.4%	55.6%	66.7%	33.3%
Active Control	10	0.29 (0.16–0.46)	70.0%	30.0%	80.0%	20.0%
Passive Control	6	0.25 (0.15–0.35)	100.0%	0.0%	100.0%	0.0%

Change in gait speed (self-selected) category after the 12-week intervention relative to baseline for patients completing the protocol as assigned (home vs community ambulation speed threshold, and Minimal Clinically Important Difference, MCID). As per the inclusion criteria, at baseline, all participants were at or below home ambulation gait speed. Post intervention, the Ekso group had the highest proportion of participants that moved into the community ambulation category (statistically significant), while Passive Control group had zero change. One third of patients (3 of 9) in the Ekso group exceeded the MCID, the highest of the three groups, 2 of 10 of the Active Control, and 0 of 6 in the Passive Control group exceeded the MCID.

*IQR* Inter Quartile Range.

Considering the midpoint assessment of the primary outcome gait speed, categorical change (>0.44 m/s) we found that 4 of 5 (80%) responders in the Ekso group had achieved the threshold change at the half-way point of the training regimen (after 6 of 12 weeks), 1 of 3 of the Active Control responders and 0 of 0 in Passive Control.

### Clinical endurance and functional balance assessment

The median distance covered in the 6MWT following the 12-week intervention was 538.0 feet (Quartile 268.0–687.3) for the Ekso Group, 346.6 feet (Quartile 219.5–711.5) for the Active Control, and 320.0 feet (Quartile 148.8–466.6) for the Passive Control representing improvements of 34%, 28%, and 18%, respectively. The median time for TUG following intervention was 26.4 s (Quartile 17.3, 53.0), for the Ekso group, 30.0 s (Quartile 26.0, 70.7) Active Control, and 46.0 s (Quartile 29.0, 64.9) for the Passive Control, representing improvements of 18.7%, 19.9%, and 12.7% respectively. Within-group and between-group differences were not significant (*p* > 0.05) for both the 6MWT and TUG measures.

### Use of assistive devices for independent walking

Change in assistive device used outside of the clinic compared to baseline was assessed each training visit. The majority of participants in both the Ekso group (8/9) and the Active Control group (7/10) showed no change in type of assistive device used throughout the duration of the protocol. Of those that did report a change, one participant in the Ekso group changed from using 1 crutch to 2 crutches between midpoint and endpoint evaluations. Three participants in the Active Control group progressed to a less restrictive assistive device between baseline and midpoint assessments with one participant regressing back to their baseline device between midpoint and endpoint. No changes in type of assistive device used were observed in the Passive Control group throughout the duration of the study.

### Tolerance and safety

Three SAEs occurred during the trial: two were urinary tract infections (UTI) unrelated to the device, and one participant in the active group was admitted to a hospital with lower extremity numbness and a UTI. The numbness was deemed by the DSMB to be “possibly related” to the Active BWSTT group. No falls occurred during training or evaluation sessions in any group. From the total sample of 45 participants (run-in plus main study participants), adverse events that were deemed “possibly” or “probably” related to the device or training include the following: 12 (8 Ekso, 4 Active) upper and lower extremity musculoskeletal issues, including orthopedic pain; 4 (3 Ekso, 1 Active) neurological issues, including increased spasticity; 6 (5 Ekso, 1 Active) skin issues; and 1 (Ekso) visceral issue. Three of these were considered severe: 2 musculoskeletal (1 Ekso, 1 Active) and 1 neurological (Ekso). Ten of these AEs were deemed “unanticipated” possibly related to Ekso training (8) or BWSTT (2). In summary, active training was generally well tolerated, with several mixed AE reported in both groups.

### Therapist workload

Results from the TLX scores showed statistically significant differences in favor of BWS training in the Frustration domain (*p* = 0.021), while all other domains were comparable (Mental, Physical, Temporal, Effort, Performance; *p* > 0.05).

## Discussion

The present randomized, controlled, multi-site clinical trial assessing intensive training using OG exoskeleton robotics (1) confirmed safe and feasible implementation in an outpatient setting, (2) found group mean increase in independent gait speed was not statistically significant, and (3) demonstrated clinically significant improvement for transition in gait speed category from home to community ambulation.

Robotic exoskeleton-based gait training is reported to have a beneficial effect on cardiovascular health in individuals with SCI [20], a potential improvement of bone health [21], and psychological benefit [22]. Evidence is controversial for training-related improvement on independent gait function as measured by walking speed [23, 24]. Our study rationale was that (a) participants with motor weakness who can physically engage in a high-dose training program, might reasonably be expected to benefit, and (b) exoskeleton robotic technologies are a practical solution to facilitate high-repetition OG gait training, and could potentially replace the manual labor component traditionally provided by therapists with inherent occupational risks.

The current study builds on existing literature that supports the safety and feasibility of intensive exoskeleton robotic training in SCI [10, 25, 26], and our results showed a statistically significant benefit for improved gait speed category associated with Ekso training. The proportion of responders above the MCID was not statistically significant, however, the Ekso group had the highest percentage of responders. We note that the MCID was conservatively set pre-trial at 0.15 m/s, however, the MCID in a population with iSCI may be considered less than half this value (0.06 m/s [27]), and our findings should be interpreted in this context. Modest improvement in functional balance (TUG) was not significant in either the Ekso or Active Control group.

Given the financial limitations of the sponsor, we recognize that this study was underpowered to prove significant effects and have calculated sample size required for a future trial based on our data. Assuming normally distributed data, a sample size of 9 in the Ekso group and 6 in the Passive Control will have 24% power to detect a difference in absolute change from baseline to endpoint evaluation for mean self-selected 10MWT between the Ekso group and the Passive Control of 0.15 m/s (the difference between the Ekso Group mean, μ_1_, of 0.18 m/s and the Passive Control mean, μ_2_, of 0.03 m/s) assuming that the common standard deviation is 0.21 m/s using a two group t-test with a 0.05 two-sided significance level. Moreover, a sample size of 32 in each group would be needed to have 80% power to detect a difference in group means of 0.15 m/s. In addition, when the sample size in each group is 32, a two group 0.025 one-sided t-test will have 81% power to reject the null hypothesis that the Ekso Group and the Passive Control are not equivalent, in favor of the alternative hypothesis that the means of the two groups are equivalent, assuming that the expected difference in means is 0.15 m/s and the common standard deviation is 0.21 m/s.

The proposed 32 participants in each study arm are consistent with recommendations in the literature, suggesting that an RCT with as few as 25 homogeneous subjects (per study arm) with a suitable Active Control may be sufficient to detect benefit of robot-assisted training [24]. With the variance of response in the active groups, we propose that an efficacy trial of this nature may be less dependent on sample size, and more on participant selection for the outcome of independent gait speed. In our sample, we aimed for participant homogeneity in a zone of enough function that one could anticipate a training-related improvement, but not so functional that robotic training would not be warranted. This criterion was insufficient to prove an average effect across the group given our small sample size, and other baseline predictive factors should be established, such that meaningful entry criteria for participants in future trials yields a more consistent treatment response.

### Use of assistive devices for independent walking

The typical outcome measure in rehabilitation for recovery of gait is gait speed (10MWT), often without regard to the assistive device used [28]. Data are compared using the same device pre to post training. However, a change to a less supportive assistive device over the course of a study protocol may itself indicate an improvement in function and may be related to gait speed, balance, independence, and/or quality of life. In this study, the 10MWT was performed with both baseline and progression of assistive devices over time (i.e. less dependence), as tolerated by the participants. Few participants reported a change in reliance on assistive devices during the protocol timeframe with the majority of participants showing no change. Varied factors may also be related to the decision to progress with respect to type of assistive device, independent of gait function. While our study showed little improvement regarding less dependence on assistive devices, we recognize that this could be an important outcome to measure in future studies. However, progression of assistive devices should follow strict a priori criteria, and inter-rater reliability of this progression should be demonstrated by the assessors.

### NASA-Task Load Index (TLX)

Task load was perceived to be similar for both therapist groups, except frustration which was significantly higher in the therapists working with the Ekso group. Scores in both groups were low, however, and the magnitude of the mean difference was small. Neither technology is likely to be frustrating when used by experienced clinicians [29].

All of the NASA-TLX dimensions, in both groups, except effort, were rated as low. These technologies may therefore result in reduced therapist workloads. Physical therapists have reported high rates of work-related pain, in part because of job tasks that require lifting and guarding patients but therapists in the current sample reported low physical demands associated with ambulation training [5, 6, 30, 31]. It should be noted, however, that only participants who could advance at least one lower extremity were included. In the case of BWSTT, advancing the lower extremities manually can result in high physical loads for therapists and increased injury frequency and severity.

The effort was the only dimension that was rated as high, where effort is a combination of mental and physical demands (both rated as low). Additional studies should seek to identify the patient and therapist factors that can influence the ratings on the NASA TLX.

### Considerations for clinical efficacy of robot-assisted gait therapy in iSCI

Foremost in consideration of treatment efficacy would be participant suitability, robot type, operation, and overall training regimen. Treatment specification is important to apply to robotic therapies as other interventions in rehabilitation medicine, where active ingredients are matched with treatment targets [32]. The overall dose is also a known influential covariate in behavioral neurorehabilitation trials. The present study showed some indication of the rate of improvement after 6 weeks (Ekso), which suggests that the overall dose should be examined systematically. As well, the long-term after-effects might be differentially affected by duration of training.

We also considered the outcome to be assessed in determining treatment efficacy, where the outcome may be matched to training specificity. Here we selected gait speed (independent and outside of the device), which is considered a gold standard in gait rehabilitation, however, some argue [33] that important treatment-related clinical improvement can be measured in other domains without change in gait speed.

Finally, how the outcome change is statistically determined, at the group or individual level is important to interpret the findings and draw conclusions. The gold standard in clinical trials is to show superiority or non-inferiority of a novel intervention group versus existing best practice. Robust effects should be demonstrated at the group level, even accounting for intragroup variability, by enrolling a sufficient sample for statistical power. However, a recent study sheds light on this approach, indicating the lack of group-to-individual generalizability [34]. The next step is to refine what constitutes who might ‘reasonably respond’, and systematically test it. Identifying features of who does not respond is equally important as to who does, so the non-responders can consider alternative interventions that improve function in other domains and ultimately quality of life.

### Limitations and recommendations for future studies

The simple randomization method in the present study resulted in non-significant statistical difference in baseline features between the three groups, however, trends in differences may be considered in the clinical interpretation of our findings. Future clinical trials should also consider (1) sufficient sample size to detect a statistical difference in group mean data, (2) anticipating a small increase in gait speed in the Passive Control group potentially a repeated assessment on the 10MWT or association with a gait clinical trial, (3) participant characteristics (clinical features) that might limit the response to this form of training, (4) restricting the number of assessments on a given day to avoid testing fatigue, and (5) including patient-reported outcome measures that detect potentially small outcome differences in domains not easily measured by the usually applied clinical assessments.

## Conclusions

We conclude that an intensive three-month course of exoskeleton robotic training in people with iSCI and limited independent gait function, can improve clinical ambulation category in a portion of participants. Further exploration of individual characteristics that predict individual-level response to intervention is needed, and may be useful for future trial inclusion criteria, as well as clinical prescription.

## Data Availability

Due to the nature of this research, participants of this study did not agree for their data to be shared publicly, so supporting data is not available.
